# The importance of improving intervention coverage measurement for ensuring all women, children and adolescents are reached with the health care services they need

**DOI:** 10.7189/jogh.10.010102

**Published:** 2020-06

**Authors:** Jennifer Requejo, Agbessi Amouzou

**Affiliations:** 1 Division of Data, Analysis, Planning and Monitoring, United Nations Children’s Fund, New York, New York, USA; 2 Department of International Health, Johns Hopkins University, Baltimore, Maryland, USA

The United Nations General Assembly adopted the Sustainable Development Goal Framework (SDGs) in September 2015 with a set of 17 goals and 169 targets to be achieved by 2030 [1]. The Global Strategy for Women’s, Children’s and Adolescents’ Health (2016-2030), in support of Every Women Every Child (EWEC GS) [2], was launched shortly afterwards to translate the broad SDG agenda into concrete guidance on how to ensure every woman, child and adolescent can realize their right to the highest-attainable standard of health. Regular monitoring of progress towards the SDG and EWEC GS goals is essential for determining where efforts need to be accelerated, where lessons can be learned and scaled up, and to hold us all to account for our roles in making universal health coverage a reality. Embedded in the EWEC GS is a monitoring framework that includes a set of indicators which cover the dimensions of survive, thrive and transform. The Countdown to 2030 indicator list complements and expands upon the EWEC GS indicator set by focusing on intervention coverage and equity in coverage. The Countdown indicators are organized into two main categories: Those that capture information about effective interventions for improving maternal, newborn, and child survival and for which comparable, high quality data are available; and those for which further measurement work is needed so that standards can be put into place for data collection, reporting and interpretation.

In addition to reporting on the first category of indicators through the Countdown country profiles and associated analyses [3], a central remit of the Countdown to 2030 Coverage Technical Working Group is to improve the measurement of this second category of core indicators on women’s, children’s and adolescents’ health. The aim of this measurement work is to increase the availability of timely data for assessing progress and for helping countries modify existing programs and develop new programs to increase coverage of high-quality interventions and ultimately improve health outcomes. This supplement presents a diverse set of secondary analyses undertaken by the Countdown to 2030 Coverage Technical Working Group. It focuses on intervention coverage and effective coverage measurement in the 81 highest maternal and child mortality burden countries. The collection of articles in the supplement build off previous analyses of intervention coverage conducted and published under the Child Health and Epidemiology Reference Group (CHERG) (http://collections.plos.org/measuring-coverage-in-mnch) and Countdown to 2015, the predecessor of Countdown to 2030, (http://www.tandfonline.com/toc/zgha20/8/s5). Each article explores measurement challenges or levels, trends and correlates of specific indicators related to women’s, children’s and adolescents’ health and nutrition, identifies strategies to increase the coverage of the interventions captured through these indicators, and suggests ways forward for addressing remaining data and research gaps.

The article by Amouzou et al [4], for example, investigates the discordance in intervention coverage for postnatal care for mothers and postnatal care for babies, finding that a large proportion of this variance is related to the current approach for measuring postnatal care which does not adequately distinguish between intrapartum and postnatal care. Suggestions are made for addressing the challenge of capturing the timing of intrapartum care compared to a first postnatal care visit. The paper authored by Jiwani et al [5] explores indicators available on the number, timing and content of antenatal care in 54 countries with a Demographic Health Survey or Multiple Indicator Cluster Survey conducted since 2012. Her analysis shows that antenatal care initiation in these countries is a key factor in the total number of visits received and is directly related to key social determinants such as women’s education level and living in rural areas. The paper from Andrus et al. [6] focusses on predictors of coverage of treatment for diarrheal diseases, which remain a leading cause of child deaths, particularly in low-and-middle income countries. Her paper finds that community-based interventions are critical for increasing coverage of oral rehydration solution as are strategies for reducing out of pocket expenditures for treatment services for childhood illnesses. The article led by Trivedi and colleagues [7] assesses country progress towards the elimination of mother to child transmission of syphilis and HIV with a focus on syphilis. The findings indicate that countries need additional support and options for affordable and integrated testing platforms to expand syphilis screening during antenatal care visits. Treatment services for women testing positive for syphilis also need to be scaled up to reduce congenital syphilis cases. Using household survey and available health facility data from Malawi, Joseph and colleagues [8] point out that although many women are accessing antenatal care, they are not receiving recommended nutrition interventions crucial for their health and the health of their babies. This study highlights the importance of linking available data sources to improve our understanding of the content of maternal health services. Sauer and colleagues tackle the challenge of estimating the variance of measures of effective coverage that combine data from multiple sources such as household and quality of care surveys. They examine the performance of three methods – the exact, delta, and parametric bootstrap methods – and formulate recommendations for the appropriate method to use based the data at hand [9].

In sum, this short collection of articles helps advance our understanding of progress in reaching all women, children and adolescents with essential health interventions, and demonstrates how the use of innovative methodological approaches to linking available data sources can shed light on gaps in the quality of care that must be addressed. Work on improving measurement and monitoring of intervention coverage needs to be supported throughout the SDG era so that we can best help countries shape programs and policies that will result in universal health coverage and lead them on the pathway to achieving the SDGs.
